# Treatability of the KMT2-Associated Neurodevelopmental Disorders Using Antisense Oligonucleotide-Based Treatments

**DOI:** 10.1155/2024/9933129

**Published:** 2024-05-29

**Authors:** Bianca Zardetto, Willeke van Roon-Mom, Annemieke Aartsma-Rus, Marlen C. Lauffer

**Affiliations:** Dutch Center for RNA Therapeutics Department of Human Genetics Leiden University Medical Center Leiden, Netherlands

## Abstract

Neurodevelopmental disorders (NDDs) of genetic origin are a group of early-onset neurological diseases with highly heterogeneous etiology and a symptomatic spectrum that includes intellectual disability, autism spectrum disorder, and learning and language disorders. One group of rare NDDs is associated with dysregulation of the KMT2 protein family. Members of this family share a common methyl transferase function and are involved in the etiology of rare haploinsufficiency disorders. For each of the *KMT2* genes, at least one distinct disorder has been reported, yet clinical manifestations often overlap for multiple of these individually very rare disorders. Clinical care is currently focused on the management of symptoms with no targeted treatments available, illustrating a high unmet medical need and the urgency of developing disease-modifying therapeutic strategies. Antisense oligonucleotides (ASOs) are one option to treat some of these rare genetic disorders. ASOs are RNA-based treatments that can be employed to modulate gene expression through various mechanisms. In this work, we discuss the phenotypic features across the *KMT2*-associated NDDs and which ASO approaches are most suited for the treatment of each associated disorder. We hereby address variant-specific strategies as well as options applicable to larger groups of patients.

## 1. Introduction

Neurodevelopmental disorders (NDDs) are a heterogeneous group of early-onset neurological diseases that include intellectual disability (ID), autism spectrum disorder (ASD), learning disorders, and language disorders [1–3]. Several thousand high-confidence and candidate NDD genes have been reported across different databases to date [4]. Until recently, available treatment options for NDDs focused on managing clinical symptoms [5], as it was thought that postnatal treatment for NDDs is unlikely to provide disease-altering effects. With more insight into the disease pathomechanisms, this common belief has been questioned [6], and there is more evidence emerging that disease-modifying therapeutic strategies for NDDs are possible. To what extent NDDs can be treated postnatally however remains to be discovered and will most likely depend on each entity individually. Examples of current developments for disease-modifying treatments of NDDs are treatments for Angelman syndrome, different types of developmental and early infantile epileptic encephalopathies (DEEs and IEEs), and neurodevelopmental psychiatric disorders [7–9].

Advances in RNA-targeting therapies are proving that antisense oligonucleotide- (ASO-) mediated treatments are one option for providing urgently needed treatments that can also be applied to NDDs [10]. ASOs can modulate gene expression through a variety of different mechanisms, like knockdown of toxic proteins or restoration of reading frames for the production of (partially) functional proteins. ASOs are particularly interesting because they can be developed in a variant-specific fashion for ultrarare disorders [11] and can also be used to treat larger groups of patients [12–15]. Treatment of larger groups of patients can mean that an ASO targets a common variant found at a high population frequency; for certain types of genetic disorders, it can also mean that one ASO alone is applicable to all individuals suffering from that particular disease (see Sections 4 and 5). To successfully develop an ASO-based, disease-modifying treatment, several steps need to be taken, such as (i) deciding which ASO approach is most suitable for the disorder in question, (ii) selecting eligible candidates for individualized treatments, (iii) defining clinical outcome measures, and (iv) setting up preclinical assays with functional readouts. Recommendations for deciding on the appropriate treatment strategy and prioritisation of eligible pathogenic variants have already been addressed by our group [16, 17], and guidelines on preclinical testing of ASOs have been established together with our group [18, 19].

By applying these recommendations and guidelines, we discuss and investigate the treatability of a rare group of NDDs associated with the dysregulation of the KMT2 family of proteins. We provide concrete examples of different potential ASO-based strategies that can be applied to this group of disorders for which no disease-modifying treatment is yet available. Both variant-specific and more general approaches—applicable to multiple individuals—are illustrated, ranging from EMA- and FDA-approved chemistries and mechanisms to experimental strategies. Furthermore, we take a new approach to identify clinical outcome measures, which are crucial for ASO developments, by comparing reported phenotypic features of the different NDDs associated with loss of function in the *KMT2* genes. We argue that overlapping clinical phenotypes within a disease group can be used to identify and define common clinical outcome measures, especially when patient cohorts are extremely small. Shared phenotypes and disease pathomechanisms within the *KMT2*-associated disorders can further contribute to establishing more widely applicable assays for preclinical development. Overall, we discuss novel targets and explore different approaches for the development of ASOs for these rare NDDs, which open new avenues for the treatment of the *KMT2*-associated disorders.

## 2. Methods

The official HUGO Gene Nomenclature Committee (HGNC) symbols are used throughout the text and figures, and variants are provided in accordance with HGVS nomenclature (https://hgvs-nomenclature.org/stable/). For the gene overview figures, the frame of exons was identified using the MANE select transcript available in the Ensembl genome browser (release 109, https://genome.ucsc.edu/index.html) [20]. The MANE select transcripts were used throughout. UniProt (release 2022_05, https://www.uniprot.org) was used to identify and locate relevant protein domains. To identify eligible variants for each of the *KMT2* genes, HGMD (Human Gene Mutation Database, accessed in July 2023, https://www.hgmd.cf.ac.uk/ac/index.php) [21], ClinVar (last accessed in June 2023, https://www.ncbi.nlm.nih.gov/clinvar/), and LOVD (Leiden Open Variation Database, version 3.0, build 29, last accessed in August 2023 https://www.lovd.nl/3.0/home) [22] were screened. Only pathogenic and likely pathogenic variants linked to a clear phenotype were considered in these searches. Further screening for published variants was performed using PubMed (https://pubmed.ncbi.nlm.nih.gov/) and the Mastermind search engine (https://mastermind.genomenon.com/). The screening and selection process for amenable variants was in compliance with the guidelines established by the Dutch Center for RNA Therapeutics [17].

## 3. KMT2-Associated Neurodevelopmental Disorders

The *KMT2* gene family consists of eight members (*KMT2A-H*), all of whom have been implicated in the etiology of NDDs. Despite being eight different genes associated with eight different disorders, the clinical manifestations of these entities show significant overlap; at the same time, each disorder maintains unique features. The *KMT2*-associated NDDs can be caused by different types of pathogenic variants and disease mechanisms, and thus, the pathomechanistic and phenotypic spectrum of this disease family offers the opportunity to investigate a wide range of suitable targets for variant-specific ASOs and ASO therapies applicable to larger groups of patients.

### 3.1. KMT2 Proteins

The histone-lysine N-methyltransferase 2 (KMT2) protein family is a group of well-conserved histone modifiers. This family of enzymes includes KMT2A (also known as MLL1), KMT2B (MLL2), KMT2C (MLL3), KMT2D (MLL4), KMT2E (MLL5), SETD1A (KMT2F), SETD1B (KMT2G), and ASH1L (KMT2H) [23]. While the proteins vary overall in domain architecture, they share a common SET domain [24], which is directly associated with their methyltransferase function. KMT2 complexes are responsible for the methylation of lysine 4 on histone H3 (H3K4), which is associated with an active transcription status [25]. The activity of the *KMT2* genes has been linked to the transcriptional activation of genes involved in several biological pathways, including (neuro-)development, differentiation, and cell cycle regulation [26]. By modulating transcriptional activation, the KMT2 proteins cover an essential role in the development of the human brain, and their methyltransferase activity is further required for its maturation throughout childhood [27, 28], suggesting an opportunity for therapeutic intervention postnatally.

At least one distinct NDD has been described for each member of the KMT2 family. The full list of genes and associated conditions can be found in Table 1.

### 3.2. Clinical Manifestations and Phenotypic Commonalities

**Figure 1:**
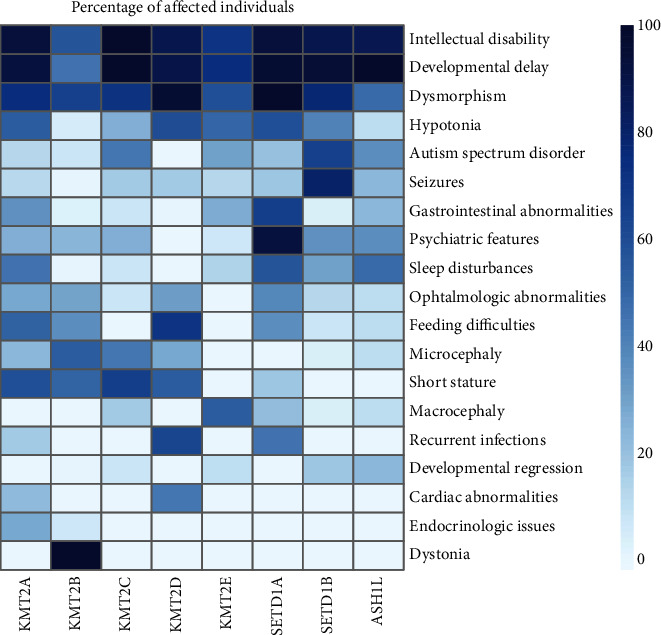
Summary of phenotypic features across the *KMT2*-associated NDDs. The intensity values represent the frequency (in percentage) with which each clinical symptom has been reported in affected individuals within the literature. The clinical signs are displayed in descending order from the most common among the gene-associated disorders to the least common. The number of cases considered for analysis varies for each gene, and it is here reported within brackets: *KMT2A* (*n* = 205), *KMT2B* (*n* = 133), *KMT2C* (*n* = 11), *KMT2D* (*n* = 359), *KMT2E* (*n* = 52), *SETD1A* (*n* = 15), *SETD1B* (*n* = 47), and *ASH1L* (*n* = 8). A summary of the published literature reporting patient phenotypes for *KMT2*-associated disorders which was used to perform our analysis can be found in Supplementary Table S2.

The phenotypic presentation of the *KMT2*-associated NDDs encompasses a spectrum of symptoms including neuronal as well as extraneuronal manifestations (Figure 1). We here present the first broad comparison of observed manifestations across these eight rare NDDs (Figure 1). By combining the case reports presented in the literature, we identified many similarities and overlapping phenotypical spectra among the *KMT2*-associated disorders. The most common features were intellectual disability (reported in 8/8 disorders, identified in 57-100% of cases per disorder), developmental delay (8/8, 47-100%), hypotonia (8/8, 7-67%), seizures (8/8, 2-79%), autism spectrum disorder (7/8, 10-64%), and gastrointestinal abnormalities (7/8, 9-67%). Furthermore, psychiatric features (7/8, 9-93%) and sleep disturbances (6/8, 9-71%) were also often noted.

### 3.3. Treatability of the Clinical Manifestations

There are some considerations to be made regarding the amenability to treatment of phenotypic manifestations, regardless of the specific ASO strategy. Neurological disorders are, at this point, the most suitable type of disease for ASO development as the organs that are predominantly affected—the brain, spinal cord, and retina—can easily be reached through local administration, e.g., intrathecal and intraventricular injections. This permits high local exposure while allowing for a lower treatment frequency, lower dosage, and lower systemic effects [60, 61]. For a disorder to be eligible for genetic treatments, clinical manifestations must be treatable and clear clinical outcome measures should be definable. ASOs can, for example, halt or slow down progressive disorders (cognitive decline or developmental regression). ASO-amenable clinical manifestations also include features that can be measured for duration or frequency, i.e., seizures, psychotic episodes, and behavioural abnormalities. Not treatable, for example, are congenital disorders and anatomical abnormalities. In line with this criteria, targetable features should be caused by the disruption of a gene that still holds a function postnatally and that should be active during treatment.

To properly define clinical outcome measures and assess treatment effects, sufficient clinical information regarding the individuals' phenotypes and the course of the disease is necessary [19]. However, information regarding the natural history and progression of rare disorders is limited and often incomplete simply on the basis of scarce accounts. Phenotypic manifestations tend to be variable, and differences can be exacerbated by the small number of recorded patients. This is also true for the *KMT2*-associated disorders, especially for diseases that have only recently been reported, such as the disorders associated with *SETD1A* [50] and *SETD1B* [54]. On the contrary, Kabuki syndrome, associated with variants in *KMT2D* [39], can benefit from decades of accumulated data.

One possible workaround to the lack of natural history data is the identification of common clinical manifestations in a group of related disorders that may provide insights into the individual conditions, as well as critical information that can be relevant to multiple patients during treatment development. We have applied this approach to the *KMT2*-associated disorders as shown in Figure 1, clearly illustrating an overlap in the clinical presentation. The clinical synopsis of the *KMT2*-associated disorders includes several symptoms for which quantifiable outcome measures can be defined and used to measure treatment effectiveness. Among these, we can highlight in particular seizures and psychotic episodes, which could be monitored during treatment for multiple of these conditions. It has also been demonstrated that the KMT2 proteins are active postnatally [27, 28, 50], which is important for possible genetic therapies. Since ASOs are particularly suitable for progressive diseases, the most ideal candidate for an ASO therapy in this group is *KMT2B*-associated dystonia 28 (DYT28). Here, it would be possible to monitor the effects of an ASO treatment on the appearance of dystonic movements and progressive degeneration in the affected individuals. Moreover, individual cases of *SETD1B-*, *KMT2E-*, *ASH1L*-, and *KMT2C*-associated disorders have been reported with speech and developmental regression [49, 62, 63], but this is not yet sufficient to make a general statement on the progressive nature of these disorders.

In summary, their ASO-amenable clinical presentation and overlapping clinical features make the *KMT2*-associated NDDs an intriguing group to study.

## 4. ASO Treatment Modalities

Among the RNA-based therapeutics, ASOs are emerging as a promising modality for treating rare genetic diseases [64, 65]. ASOs are small, single-stranded pieces of chemically modified nucleic acids that can target sequences in the (pre-)mRNA through Watson-Crick base pairing and modulate gene expression through a variety of different mechanisms [47]. ASOs can be developed to aid multiple (even up to all) individuals affected by a specific disorder (generalized approaches) and can also be adapted to individual, variant-specific cases (Table 2).

There are two commonly used mechanisms of action to regulate and interact with the (pre-)mRNA transcript: steric blocking and reduction via RNase H [73].

### 4.1. Steric Blocking

One way to employ ASOs for disease-modifying therapies is through a mechanism called steric blocking. Here, the ASOs bind to regulatory sequences within the (pre-)mRNA and block the interaction of these sequences with their physiological interactors, for example, elements of the splicing apparatus and microRNAs [10].

Steric blocking ASOs can be used to modulate splicing, and these specific ASOs are referred to as splice-switching ASOs (ssASOs). ssASOs bind to different regulatory sequences along the pre-mRNA and can lead to the skipping/exclusion of exons during the splicing process or the inclusion of an exon. ssASOs can fully or partially rescue protein function by restoring the open reading frame or by removing exons that contain toxic gain of function variants. Depending on the desired effect, the regulatory sequences that can be targeted with ssASOs are the are the 5′ donor site, 3′ acceptor site, the branch point [74], and intronic and exonic splice enhancers and silencers.

Steric blocking ASOs can also target regulatory sequences and structures in the untranslated regions (UTRs). This interaction can stabilize transcripts and increase target protein levels [75, 76]. Finally, steric blocking ASOs can also be employed to prevent the translation of a transcript by the ribosomes, reducing protein levels [77, 78].

### 4.2. RNase H-Mediated Degradation

The second ASO mechanism of action that can be employed to regulate gene expression is the RNase H-mediated degradation of RNA transcripts. ASOs that can recruit RNase H to (pre-)mRNA transcripts are called gapmer ASOs [79, 80]. These ASOs contain a gap in the center that maintains DNA properties. The binding of these gapmer ASOs to (pre-)mRNA creates DNA:RNA hybrids which the RNase H enzyme recognizes and cleaves [81, 82]. This ultimately leads to (pre-)mRNA degradation and results in a knockdown of transcript and protein levels.

Counterintuitively, gapmer ASOs can also be applied to increase protein expression. RNase H-mediated degradation can be directed towards naturally occurring antisense transcripts (NATs), which are RNA transcripts transcribed from the opposite strand of the gene of interest on the DNA. Transcription of these antisense transcripts can block or reduce transcription of the sense transcript—the gene of interest. By targeting NATs with a gapmer ASO, this competitive transcription is reduced and more transcripts from the gene of interest can be generated, ultimately increasing protein levels [83, 84].

## 5. Treatment Approaches and Considerations for the KMT2-Associated Disorders

No disease-modifying, genetic treatments are yet available for any of the *KMT2*-associated disorders, and most therapeutic strategies focus on managing clinical manifestations and preventing secondary complications [32, 34, 40, 85]. Yet, based on the clinical presentation, the *KMT2*-associated NDDs can potentially benefit from ASO approaches, which has already been suggested by others [86–88].

ASOs can be used in different ways to treat the *KMT2*-associated disorders, as variant-specific treatments or therapies for larger groups of patients. In the following, we discuss the different strategies and how they can be applied to each of the genes and related disorders. The proposed therapeutic approaches have been summarized in Table 3.

### 5.1. Steric Blocking ASOs

Steric blocking can be employed to modulate splicing (ssASOs) or regulate transcript stability as described in Section 4.1. We provide concrete examples that illustrate for which of the *KMT2* genes each of the approaches is applicable.

#### 5.1.1. Exon Skipping

Exon skipping is the process by which ssASOs induce the removal of coding sequences (exons) from a pre-mRNA transcript during the splicing process [93]. The skipping of canonical exons has been successfully used to develop several market-approved drugs [60].

**Figure 2:**
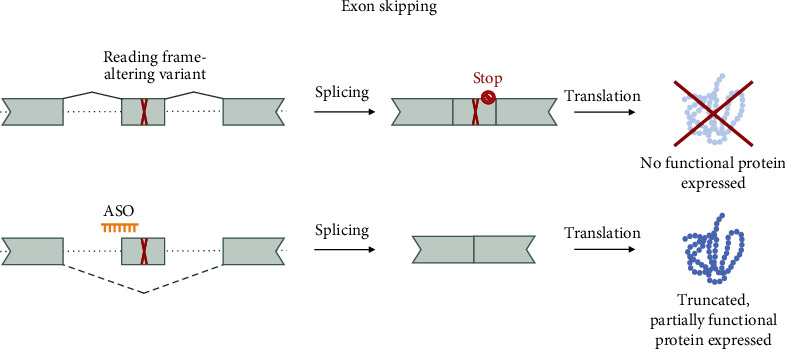
Splice switching ASOs for exon skipping. ssASOs can interact with specific regulatory sequences and modulate splicing, leading to the removal of exonic sequences. In the case of nonsense variants and small indels leading to a frameshift and early truncation, this mechanism can be exploited to restore the reading frame. This can result in the production of an internally truncated protein that still maintains function. Splicing events are indicated by conical connecting lines, full for the canonical splicing, and dashed for exon skipping.

This approach is especially suitable for disease-causing, exonic variants such as nonsense, frameshift, and, in rare cases, pathogenic missense variants. Exon skipping can lead to the production of an (internally) truncated, partially functioning protein by restoring the reading frame (Figure 2). Note that it is crucial to establish preclinically that the proteins encoded by mRNAs lacking the skipped exons are indeed (partially) functional.

Even though ssASOs for canonical exon skipping do not necessarily bind directly to the variant site, they can be customized to skip an exon containing an individual's specific variant. This is particularly useful for ultrarare disorders where only a few cases are reported.

**Figure 3:**
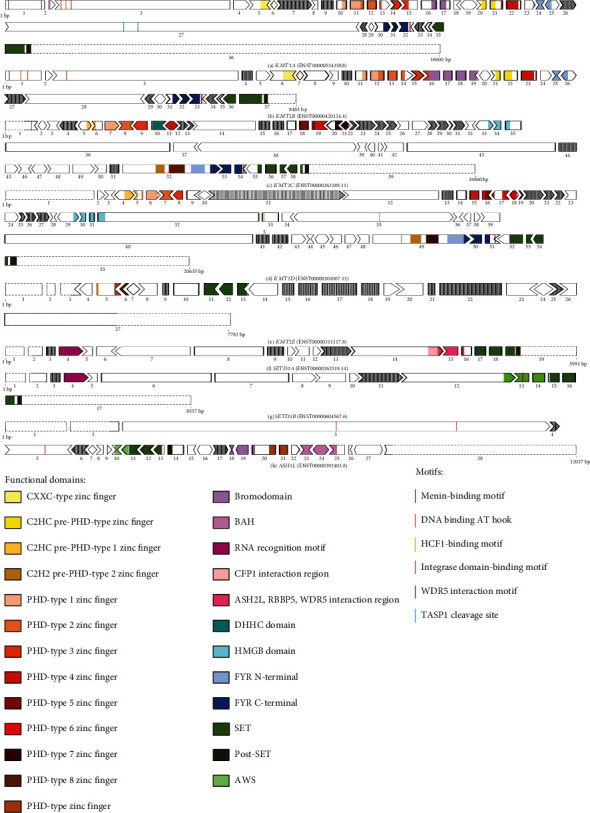
Schematic representation of *KMT2* genes highlighting functional domains and frame of exons screened for an exon skipping approach in this study. Shared domains are indicated with the same color across the panels, as indicated in the legend. The shape of the exon indicates the frame and the size of the exons is to scale. In-frame exons are additionally marked by thicker outlines. Exons amenable to exon skipping can be identified by vertical stripes. The label underneath the exons is the exon number according to the reported NM transcript. The space dividing the exons, representing the intronic sequences, is not to scale. A continuous line demarks coding regions, while dotted lines represent the untranslated regions. The complete list of screened variants that are amenable to an exon skipping approach can be found in Table S1.

Through the screening of pathogenic variants present in publicly available databases, we identified several potential targets for a personalized exon skipping approach for the *KMT2*-associated disorders. Amenability was assessed according to the selection guidelines established by the Dutch Center for RNA Therapeutics. We refer to the guidelines for the detailed description of the selection process for amenable variants [17]. Briefly, canonical exons should be in-frame (exon length divisible by 3), short (less than 10% of coding region), and not contain important functional domains. Thus, possible targets with specific variants include exon 9 of *KMT2A* (containing NM_001197104.2:c.4177dup, p.Ile1393Asnfs∗14), exon 12 of *KMT2C* (NM_170606.3:c.1690A>T, p.Lys564Ter), exon 13 of *KMT2D* (NM_003482.4:c.3968dup, p.Gly1323_Arg1324insTer), exon 15 of *KMT2E* (NM_182931.3:c.1646_1650del, p.Ile549fs), and exon 17 of *ASH1L* (NM_018489.3:c.7261C>T, p.Arg2421Ter) (Supplementary Table S1 and Figures 3(a), 3(c), 3(d), 3(e), and 3(h)). The full list of exons suitable for this approach and the contained variants are reported in Table 3 and in Table S1, respectively.

The canonical exon skipping approach can also be used to skip exons containing pathogenic variants that affect more than one patient. An overview of all currently known pathogenic variants and their estimated prevalence for a given gene can indicate which exons are most suitable for such an approach and the number of patients that could potentially benefit. Examples for this therapeutic strategy are the small exons 41 and 42 of the *KMT2D* gene (Figure 3(d)). Several patients with Kabuki syndrome, carrying nonsense and frameshift variants within these two exons, have been reported (Table S1). Additional exons carrying multiple frameshift and nonsense variants which would be good candidates for canonical exon skipping are exon 27 of *KMT2B*; exon 44 of *KMT2C*; exons 20 and 21 of *KMT2D*; exons 16, 17, 18, 22, and 25 of *KMT2E*; and exon 9 of *SETD1A* (see Table 3 and S1 for the complete list of amenable targets). Exons fulfilling the criteria for canonical exon skipping are also highlighted in Figure 3.

It is currently not possible to extrapolate the true percentage of variants and patients that can be targeted with an exon skipping approach. This is partly because many variants are not reported, and information regarding the patient's phenotype and status is limited. Furthermore, the diagnostic yield is currently limited to about 50%, meaning many affected individuals do not receive a diagnosis and we are missing in particular variants outside of the coding regions [94, 95]. This especially affects a subcategory of exon skipping, in which ssASOs are employed to correct so-called cryptic splicing. Cryptic splicing is a naturally occurring process by which the splicing apparatus uses splice sites (cryptic splice sites) outside of the canonical splice sites, creating transcripts that can incorporate parts of introns (then called cryptic exons) or removing parts of canonical exons [74, 96, 97]. Certain pathogenic variants can strengthen or create new cryptic splice sites, resulting in increased usage of these sites for the generation of mRNA transcripts. Usage of these cryptic splice sites mostly leads to a disruption of the reading frame and a reduction in the amount of functional protein produced. ssASOs can bind to the cryptic splice sites (exonic and intronic) and hide them from the splicing apparatus to restore canonical splicing [98, 99]. These variants are the ideal targets for ssASOs but are less likely to be picked up with routine diagnostic sequencing and rarely have their effect reliably predicted. While no target variant has yet been identified, cryptic exon skipping is universally applicable for the KMT2 disorders.

Exon skipping can also be employed to treat toxic gain of function (GoF) and dominant-negative variants. Either a knockdown of the full transcript can be achieved by skipping any out-of-frame exon, or the variant-containing exon can be removed independent of the reading frame. In the context of haploinsufficiency, it is possible to design allele-specific ssASOs [100], which are used to modify only variant-containing alleles. The knockdown approach is best suited if the gene is tolerant to loss of function; nevertheless, it could be used to partially reduce expression levels. Therefore, this knockdown strategy could also be considered with caution for haploinsufficiency conditions such as the *KMT2*-associated diseases.

#### 5.1.2. Targeting Naturally Occurring Nonproductive Alternative Splicing Events

ssASO can also modulate naturally occurring nonproductive alternative splicing (AS) events with the overall aim of increasing levels of the canonical transcript and, consequently, the endogenous protein [90]. These AS events include cassette exons, specifically poison exons, alternative 5′ and 3′ splice sites, and alternative introns (exitrons). While many of the AS events occur to create a larger variety of transcripts and are often necessary for development [101], we here only address AS events that lead to nonproductive transcripts that can be modified with ssASOs to generate the canonical transcript that encodes the functional protein.

**Figure 4:**
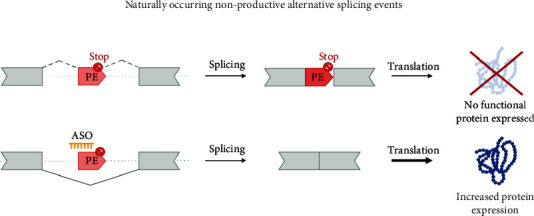
Splice switching ASOs to target naturally occurring nonproductive AS events. The ability of ASOs to modulate splicing and induce exon skipping can be harnessed to remove alternative exons which contain premature stop codons. By suppressing the inclusion of these exons, it is possible to increase the canonical transcript and the production of a normal, functional protein. Alternative splicing is indicated by dashed lines. PE: poison exon.

Poison exons occur naturally and are highly conserved alternative exons. Their inclusion in the transcript will lead to nonsense-mediated decay, as the poison exon contains a premature termination codon [102]. In recent years, there has been an increase in the identification of such transcripts and background splicing events in general, and more of these events may be uncovered in the future [103]. Forgoing the nonproductive AS events through the use of ssASOs generally leads to higher levels of protein-coding transcripts (Figure 4). Any disease, including the *KMT2*-associated disorders, for which the pathomechanism is based on haploinsufficiency, is ideally suited for such an approach.

For the *KMT2*-associated disorders, several nonproductive AS events have been identified and could be used as putative targets for generalized treatment approaches (Table 3). This is the case for *KMT2A*, *KMT2B*, *KMT2C*, *KMT2E*, and *ASH1L*, where poison exons have recently been reported [89, 90, 92]. Not all of the PEs have been reported consistently across different publications; thus, it is necessary to confirm the presence and abundance of these alternative events in the affected tissues, which in the case of the *KMT2*-associated disorders is the central nervous system. Whether it is possible to upregulate the canonical transcript to the level needed to rescue the patients' phenotypes remains a main concern for this approach.

#### 5.1.3. Targeting of Regulatory Elements within the UTRs

Targeting regulatory elements within the 5′ and 3′ UTRs is another way to modify protein levels. Steric blocking of translation inhibitory elements including the upstream open reading frames (uORFs) and structured regions has been shown to increase protein levels [104–106]. Stabilizing otherwise fragile transcripts by targeting the UTRs is also possible [107]. For the *KMT2*-associated NDDs, using this method can be used to increase the protein levels produced from the wild-type (WT) transcript.

**Figure 5:**
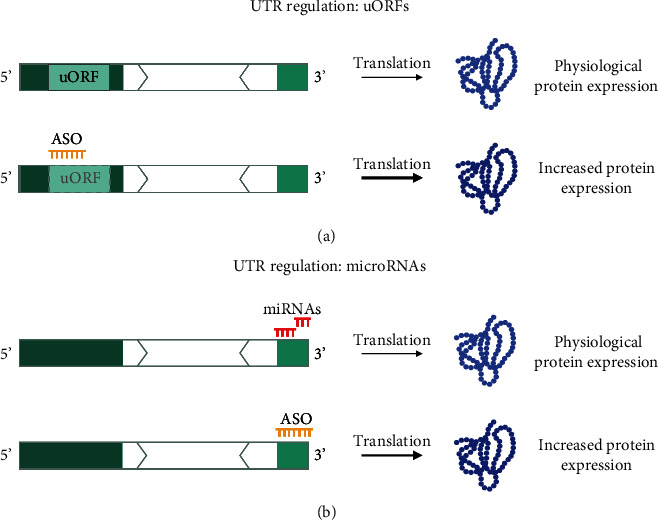
Steric blocking ASOs to target regulatory elements in the UTRs. Steric blocking ASOs can stabilize target transcripts by binding to the 5′ or 3′ UTRs. (a) In the 5′ UTR, ASOs can be used to bind to uORFs or structured regions to increase the translation of the primary ORF. (b) In the 3′ UTR, ASOs can interfere with degrading complexes and increase half-life. Overall, this application of steric blocking ASOs binding to the UTRs means to increase protein levels compared to physiological protein expression. uORF: upstream open reading frame; miRNA: micro-RNA.

uORFs are ORFs situated within the 5′ UTR of a transcript [108]. They have been shown to play a regulatory role in the translation of the primary ORF (pORF) [76]. Often, uORFs reduce the translation from the pORF, affecting the levels of protein produced. ASOs offer the opportunity to target these uORFs and increase the protein levels without disrupting the canonical mRNA. However, not all uORFs are necessarily inhibitory and can encode polypeptides with separate functions. The exact effect of these uORFs should be ascertained to ensure that interference via ASO has the desired effect [70]. Although no ASO based on this strategy has yet been approved as a drug, this approach has already been tested for several genes *in vitro* and *in vivo* [76, 106]. To date, *KMT2C*, *KMT2D*, *KMT2E*, and *ASH1L* have been reported to contain uORFs [91, 92] (Table 3). Regulation of these elements using steric blocking ASOs could be employed to increase the levels of the WT protein of the respective disorder and improve the phenotype (Figure 5(a)).

Steric blocking ASOs can also be applied to target other UTR regulatory elements, such as structured regions. The formation of secondary structures in the 5′ UTR mainly has an inhibitory effect on translation initiation, although specific structures can also have the opposite effect [109]. ASOs hybridizing with these structures have been demonstrated to reduce their inhibitory effect and increase protein levels in different genes [104, 105].

ASOs can further increase the half-life of mRNA transcripts by binding to either UTR and plausibly protect these sequences from degrading complexes. In particular, ASO-binding can interfere with miRNAs targeting the 3′ UTR, contributing to transcript stabilization (Figure 5(b)) [110].

### 5.2. Gapmer ASOs

The second category of ASOs regards those that recruit RNase H to induce the knockdown of the target transcript (see Section 4.2). Since our focus is the possible treatment of the *KMT2*-disorders, which are known to be haploinsufficiency conditions, we here consider only the applications that lead to upregulation of the WT allele and exceptions for toxic GoF variants.

#### 5.2.1. Naturally Occurring Antisense Transcripts

**Figure 6:**
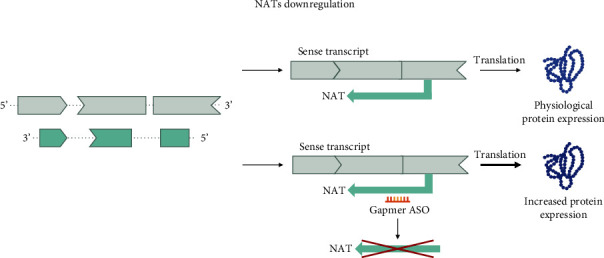
Gapmer ASOs for naturally occurring antisense transcripts. Gapmer ASOs can direct the enzymatic action of RNase H towards NATs that interfere with the transcription of the canonical transcript. By downregulating these antisense RNA molecules, it is possible to increase the levels of functional protein produced. NAT: naturally occurring antisense transcript.

Regulating the levels of NATs is another means of influencing the level of protein-coding transcripts. NATs are RNA molecules transcribed from the antisense strand of the DNA and can interfere with transcription on the sense strand containing the gene of interest (Figure 6). These regulatory RNA molecules have been associated with a wide variety of physiological and pathological processes [111]. By downregulating specific NATs with gapmer ASOs, it is possible to achieve the upregulation of the gene of interest from the sense strand [112].

Especially disorders associated with haploinsufficiency could benefit from this application, which will lead to the upregulation of protein produced by the unaffected WT transcript. In the case of the *KMT2*-associated disorders, three antisense transcripts, namely, *KMT2A* antisense RNA 1 (*TTC36-AS1*), *KMT2E-AS1*, and *ASHL1-AS1*, have so far been identified and could serve as possible targets [92] (Table 3). However, the presence of these long noncoding RNAs alone does not ensure that an ASO treatment would be efficacious for the associated disorders. For example, NAT transcription is tissue-dependent [113], and the NATs might not perform their function in a disease-affected cell type. NATs can further modulate the levels of protein-coding transcripts through various mechanisms [114], and their functional characterization remains challenging. For example, *KMT2E-AS1* has been shown to have a stabilizing effect on the KMT2E protein, and its downregulation promotes hypoxic endothelial pathology in pulmonary hypertension [115]. This indicates that while present, this NAT might not be a suitable target to treat the haploinsufficiency disorder associated with *KMT2E*.

#### 5.2.2. Toxic Gain of Function and Dominant Negative Variants

Gapmer ASOs that recruit RNase H to induce the degradation of target transcripts are ideally suited to tackle pathogenic GoF variants and copy number gains in genes that tolerate loss of function [16]. Also, variants leading to a dominant negative effect can benefit from RNase H-mediated knockdown. In the case of heterozygous variants, this treatment modality can be customized to exclusively target the variant-carrying allele, while still allowing the translation of the WT allele. Notably, ASO-induced degradation is variable and often incomplete [116]; therefore, residual expression of toxic proteins is likely to persist after treatment. This is particularly relevant in the case of dominant negative variants and calls for careful consideration when estimating knockdown efficacy and treatment benefit.

Interestingly, rare cases of alleged GoF mutations have been reported in the *KMT2*-associated disorders [48, 117] (Table 3). These variants appear to alter the age of onset or the affected tissue, but no functional studies have been performed to confirm their effect. Although it would, in principle, be possible to consider allele-specific transcript knockdown for these GoF cases, it is challenging to estimate the extent to which it would be feasible to ameliorate the patient's phenotype. The NDDs associated with the *KMT2* family of genes are autosomal dominant disorders associated with haploinsufficiency, meaning that it is not possible to produce an adequate amount of functional protein from one allele alone to maintain physiological function [118]. The *KMT2*-associated disorders are thus not ideal candidates for gapmer ASO strategies.

Here, we illustrated that different ASO-based treatments can be considered for the *KMT2*-associated disorders. To our knowledge, no ASO treatment is under development for any of these diseases at this point. To ensure successful treatment development, further studies are required to assess the possibility of targeting the reported alternative splicing events and to confirm the function of the regulatory sequences and antisense transcripts. As of now, exon skipping for the rescue of the reading frame appears to be the most applicable strategy for this group of disorders.

## 6. Preclinical ASO Development

Once a candidate genetic variant or general target site is identified, several steps have to be taken for the successful preclinical development of an efficacious ASO [18, 119]. The drug development process for individual cases and small groups of patients is quite distinct from the traditional drug development pathway used to develop treatments for larger groups. The differences are not only cost and time related but also involve the assays that are being performed, specifically with respect to animal models.

The development process for individualized ASO treatments starts with designing and confirming the effect of the ASO at the transcript level as well as at the protein level. For all experimental designs, it is crucial to take along appropriate controls, such as scrambled ASOs [18, 119]. At this step, ideally, patient-derived cells expressing the target transcripts are being employed. This can pose a problem for neurological disorders in particular, since easily accessible cells like skin fibroblasts and lymphocytes only express 60-70% of OMIM-listed genes [120] and might not express the target transcript. It might thus be necessary to use induced pluripotent stem cells to generate the desired cell type or use methods of transdifferentiation [121]. Ultimately, ASO development can only be deemed successful following *in vitro* functional assays to validate the restoration of protein function or reduction of toxic gain of function after treatment. Functional evaluation is specific to the target gene and is preferably carried out in disease-relevant cell types. These functional assays will have to be established for a single protein or group of proteins. The assays can depend on direct read-outs, such as enzymatic function, or indirect/downstream read-outs such as effects on pathways. To identify suitable read-outs, the function of the proteins involved and the pathophysiology of the disease in question should be well enough understood. For extremely rare diseases, this will pose difficulties.

The candidate compounds should further be tested for tolerability *in vitro* and *in vivo*, and a short safety-pharmacology study should be performed *in vivo*. The current recommendation for the *in vivo* safety study is the use of WT rats to test for immediate toxic effects following intrathecal injections [18, 19]. Since the preclinical development of these ASOs is a short process that does not include animal disease models, it is challenging to assess long-term effects, and potentially, adverse effects can be missed [68, 71]. It should be carefully considered whether the potential benefits of treatment outweigh these side effects as well as the burden of the treatment. The exact requirements for the preclinical development of these individualized treatments also depend on the respective regulatory bodies, such as FDA and local bodies in European centers where named patient treatment can be employed.

For the preclinical development of ASO therapies for the *KMT2*-associated disorders, ASO screening can easily be performed in patient-derived cultured fibroblasts [122, 123]. On the other hand, functional studies of the compounds, are ideally performed in neuronal cells. Therefore, patient-derived fibroblasts and blood can be used to generate induced pluripotent stem cell-derived neuronal cultures [124–126]. As mentioned above, the genes' functions should be taken into account for developing suitable read-outs to study treatments in the *KMT2*-associated disorders. The KMT2 protein family regulates transcription through methylation; thus, the *KMT2*-associated disorders are characterised by the disruption of the methylation marker deposited by the respective KMT2 protein. Since it is not fully understood whether methylation marks can be used as an indication of the restoration of protein function after treatment, other read-outs need to be established. Assessments of neurodevelopmental phenotypes and their corrections as well as epileptogenic phenotypes manifested through altered electrophysiological characteristics can be studied in 2D and 3D neuronal networks. For example, calcium imaging and multielectrode array recordings can be performed on these 2D/3D systems and can be used as common readouts applied to all investigated proteins [127, 128]. Analyses could also include the use of cellomics to study cell morphology and changes thereof in more detail [129, 130].

In general, ideas for suitable read-outs can be taken from studies explaining the pathophysiology of a disorder, including disease hallmarks. A potential change towards a wild-type phenotype of one or more of these hallmarks can then be seen as a benefit of the treatment.

## 7. Concluding Remarks

ASO-based treatments are emerging as therapeutic options for an increasing number of rare genetic disorders, including NDDs [16, 86, 87]. The molecular pathomechanism and phenotypic presentation of the *KMT2-*associated NDDs make them attractive candidates to be considered for ASO strategies. In general, several reports support the notion that the *KMT2*-associated NDDs would benefit from ASO treatments [86, 87].

Here, we discussed and proposed at least one treatment option, either variant-specific or generalized, for each *KMT2*-associated disorder, highlighting the ample applicability of ASO-mediated approaches for this group of diseases (Table 3).

Another ASO-based strategy that could also be employed but was not explicitly explored in this work is to restore gene function by modulating pseudogenes as done for spinal muscular atrophy [68, 70]. This approach requires in-depth knowledge regarding the overlap in function of the two genes to understand which ASO strategy would be most beneficial. For example, there are five known pseudogenes for *KMT2C* (*KMT2CP1*, *KMT2CP2*, *KMT2CP3*, *KMT2CP4*, and *KMT2CP5*), but at the moment, we lack the information necessary to determine whether they would be a suitable therapeutic target.

In this study, we solely explored the treatability of the *KMT2*-associated disorders using ASO-based approaches; however, the use of ASOs is not the only possible therapeutic option for the *KMT2*-associated disorders, and other genetic strategies can be considered. For example, small-activating RNAs (saRNAs) have been proposed as a future therapeutic application for these histone-methyltransferase-associated NDDs to obtain target gene upregulation [88].

Other gene-based therapeutic strategies, such as gene replacement or gene editing, are also available for genetic NDDs [8]. For example, a CRISPR/Cas9 gene therapy treatment on patients' cells for Kabuki syndrome (ClinicalTrials.gov Identifier: NCT03855631) is being tested, although no results have been published.

It is important to note that while these genetic treatments are defined as disease-modifying, it is unlikely that they could rescue function that has already been lost and cannot correct congenital defects. However, given the neural plasticity of the brain, early treatment could have potential benefits also for nonprogressive neurodevelopmental disorders.

We also highlighted the relevance of comparing cohorts of patients with related rare disorders to obtain information regarding clinical manifestations, outcome measures, and preclinical therapy development (see Sections 3.3 and 6). Performing natural history studies is essential to help fill the gap in our understanding of the pathologies and potential progression of symptoms. Implementing the information from these studies will allow us to define realistic expectations of benefit for each potential patient and establish a window of opportunity for treatment.

In conclusion, the *KMT2*-associated disorders could benefit from ASO-based therapeutic strategies; however, each approach should be considered carefully for feasibility and benefit. As our understanding of these diseases advances and more cases are reported, the number of amenable targets and treatable individuals will likely increase.

## Figures and Tables

**Table 1:** Overview of the *KMT2* genes and the related disorders. The table summarizes information regarding the genes, such as location, and the associated NDDs.

Gene	Chromosome	∗OMIM	Disorder	#OMIM	Symbol	References
*KMT2A*	11q23	159555	Wiedemann-Steiner syndrome	605130	WDSTS	[29]; [30]; [31]; [32]
*KMT2B*	19p13	606834	Childhood-onset dystonia 18	617284	DYT28	[33]; [34]; [35]
*KMT2C*	7q36	606833	Kleefstra syndrome 2	617768	KLEFS2	[36]; [37]; [38]
*KMT2D*	12q13	602113	Kabuki syndrome 1	3147920	KABUK1	[39]; [40]; [41]; [42]; [43]; [44]; [45]; [46]; [47]
*KMT2E*	7q22	608444	O'Donnell-Luria-Rodan syndrome	618512	ODLURO	[48]; [49]
*SETD1A*	16p11	611052	Neurodevelopmental disorder with speech impairment and dysmorphic facies Early-onset epilepsy disorder Schizophrenia	619056 618832 181500	NEDSID EPEDD SCZD	[50] [51] [52]; [53]
*SETD1B*	12q24	611055	Intellectual developmental disorder with seizures and language delay	619000	IDDSELD	[54]; [55]; [56]
*ASH1L*	1q22	607999	Intellectual development disorder-52	617796	MRD52	[57]; [58]; [59]

∗ indicates the gene, # refers to a descriptive entry, in this case the specific disease/syndrome.

**Table 2:** Overview of treatment modalities and their application for variant-specific (individualized) and generalized ASO-based approaches. The table provides a short explanation of what each approach can be used for, the advantages and disadvantages of each approach, and an example of an approved ASO or ASO under development.

	Application	Variant-specific	Generalized	Explanation	Pros	Cons	Examples
Steric blocking	Exon skipping and inclusion	✓	✓	Splice modulation can be used to skip specific cryptic or variant-containing exons to restore the reading frame and lead to the translation of a (partially) functional protein. It is also possible to skip exons to achieve NMD (for KD approaches). Exon inclusion can be achieved for individual cases or larger groups of patients.	Exon-skipping of cryptic exons can restore canonical transcripts for single individuals and more common variants for groups of patients. Larger groups of patients can benefit from canonical exon skipping.	Canonical exon-skipping requires thorough analysis of left-over protein function. For AD disorders, canonical exon skipping can potentially negatively impact the intact wild-type transcript. Exon inclusion is very challenging to achieve.	Variant-specific ASOs: milasen [11] and atipeksen [67] Disease-specific ASO: nusinersen for spinal muscular atrophy [68]
	Nonproductive AS events	X	✓	By targeting nonproductive alternative splicing events, it is possible to increase levels of the canonical transcript.	Can be applied in AD disorders in which the mutant allele cannot be rescued. Applicable to larger groups of patients.	Method not applicable to all genes. The transcripts leading to nonproductive splicing need to be expressed at a sufficient level in the target tissue/cell type.	Poison-exon skipping in Dravet syndrome (*SCN1A*) [69]
	UTR targeting	✓	✓	The UTRs can be targeted to up- and downregulate transcripts through the interaction with regulatory elements.	Applicable to a variety of diseases and genetic backgrounds, e.g., haploinsufficiency disorders and also toxic GoF.	Requires knowledge of the exact effect of the regulatory sequences.	Disease-specific targeting of the 5′UTR of *SMN2* for spinal muscular atrophy [70]

RNase H-mediated KD	Knockdown sense transcript	✓	✓	Recruitment of RNase H can be used to knockdown sense transcripts in case of toxic GoF and DN effects. It can also be achieved by targeting the UTRs.	Knockdown of specific transcripts can be performed variant-specific, allele-specific, or generalized for all transcripts of a gene.	Cannot be applied to haploinsufficiency disorders. Achieving allele selectivity is challenging.	Disease-specific gapmer ASO: tofersen for *SOD1*-associated amyotrophic lateral sclerosis [71]
	Knockdown antisense transcript	X	✓	Upregulation of a transcript can be achieved by RNase H-mediated knockdown of its antisense transcript.	Approach applicable to larger groups of patients and especially for AD with haploinsufficiency.	KD of AS transcript needs to be sufficient to rescue protein levels in target tissue. Role of AS transcript needs to be known not to cause adverse effects.	Targeting of the antisense transcript of *UBE3A* for Angelman syndrome [72]

AD: autosomal dominant; DN: dominant negative; GoF: gain of function; KD: knockdown; NMD: nonsense-mediated decay; AS: alternative splicing; UTR: untranslated region.

**Table 3:** Overview of the different ASO approaches applicable to each KMT2 gene. The table provides at a glance a summary of which approach could be applied to the different genes, divided between steric blocking and RNase H-mediated degradation, and highlights specific targets for each. Exon numbers refer to the reported transcripts.

Gene	Transcript	Steric blocking			RNase H	
		Exon skipping	Nonproductive AS	UTR regulation	NATs	Toxic GoF
*KMT2A*	ENST00000534358.8	7, 9, 26, 28, 33	PE ¶	X	AS1	X
*KMT2B*	ENST00000420124.4	4, 10, 27, 29, 34	PE ¶	X	X	GoF
*KMT2C*	ENST00000262189.11	4, 12, 13, 15, 16, 17, 22, 23, 24, 25, 28, 29, 30, 31, 36, 43, 44, 51	PE (intron17) ǂ	⊢	X	X
*KMT2D*	ENST00000301067.12	11, 13, 19, 20, 21, 22, 25, 26, 27, 41, 42	X	⊢ (not MANE)	X	X
*KMT2E*	ENST00000311117.8	9, 15, 16, 17, 18, 21, 22, 25	PE (intron 21) ∗¶ǂ	uORF ¶⊢	AS1	GoF
*SETD1A*	ENST00000262519.14	3, 8, 9, 13	X	⊢	X	X
*SETD1B*	ENST00000604567.6	3, 6, 7, 11	X	⊢	X	X
*ASH1L*	ENST00000392403.8	4, 6, 17	PE (intron 24) ∗¶ǂ	uORF ¶⊢	AS1	X

AS: alternative splicing; PE: poison exon; UTR: untranslated region; NATs: naturally occurring antisense transcripts; GoF: gain of function. The additional symbols indicate in which publications the features have been reported: ǂ, Felker et al. [89]; ∗, Lim et al. [90]; ⊢, Liu et al. [91]; and ¶, Mittal et al. [92].

## Data Availability

All analyzed variants are accessible through the databases described in the Methods and supplementary material.

## Supplementary Materials

Supplementary 1 Table S1: list of (likely) pathogenic variants amenable to an exon skipping approach listed for each gene.

Supplementary 2 Supplementary methods: annotation of Supplementary Table S1 and details of the variant evaluation procedure. It includes Supplementary Table S2: summary of the analyzed published literature reporting phenotypic characteristics for the *KMT2*-associated disorders.
