# Supplementary material for: Treatability of the KMT2-Associated Neurodevelopmental Disorders Using Antisense Oligonucleotide-Based Treatments
Source: Hum Mutat. 2024 May 29;2024:9933129. doi: 10.1155/2024/9933129 (PMC11925151; doi:10.1155/2024/9933129)
Supplement: Supplementary 1 — Table S1: list of (likely) pathogenic variants amenable to an exon skipping approach listed for each gene. [file 9933129.f1.pdf]

ENST00000534358.8 (MANE select)

**Exon #**	**Frame In/out**	**Ex size in amino acids**	**Domains**	**Reference #**	**NM_001197104.2(*KMT2A*)**
1	out		Menin binding motif; integrase domain-binding motif 1		
2	out		integrase binding motif 2		
3	out		A+T hook 1, 2, 3		
4	out				
5	out		CXXC-type		
6	out		CXXC-type		
**7**	in	126 AA		1	c.3646A>T (p.Lys1216Ter)
				2	c.3651dup (p.Lys1218fs)
				3	c.3651_3652del (p.Lys1219fs)
				4	c.3680_3683del (p.Asp1227fs)
				5	c.3724C>T (p.Gln1242Ter)
				6	c.3740_3741del (p.Ser1247fs)
				7	c.3790C>T (p.Arg1264Ter)
				8	c.3809del (p.Lys1270Argfs*Ter86)
				9	c.3837del (p.Pro1281Leufs*75)
				10	c.3853C>T (p.Gln1285Ter)
				11	c.3876del (p.Lys1293Serfs*63)
				12	c.3889C>T (p.Gln1297Ter)
				13	c.3902_3905dupCAGC (p.Leu1303Serfs*24)
				14	c.3920dup (p.Gln1308fs)
				15	c.3929_3930delinsA (p.Pro1310Glnfs*46)
				16	c.3935_3936del (p.Thr1312fs)
8	out				
**9**	in	44 AA		17	c.4171C>T (p.Gln1391Ter)
				18	c.4189G>T (p.Gly1397Ter)
10	in	38 AA	PHD-type 1		
11	in	49 AA	PHD-type 1; PHD-type 2		
12	in	32 AA	PHD-type 2		
13	out		PHD-type 2		
14	in	41 AA	PHD-type 3		
15	out		PHD-type 3		
16	in	58 AA	Bromodomain		
17	in	37 AA	Bromodomain		
18	out				
19	out				
20	out		C2HC pre-PHD-type		
21	in	46 AA	C2HC pre-PHD-type; PHD-type 4		
22	in	53 AA	PHD-type 4		
23	out		FYR N-terminal		
24	out		FYR N-terminal		
25	out		FYR N-terminal		
**26**	in	62 AA			
27	out		TASP1 cleavage site 1, 2; 9aaTAD		
**28**	in	27 AA			
29	out				
30	in	57 AA	FYR C-terminal		
31	in	25 AA	FYR C-terminal		
32	out		FYR C-terminal; WRD5 interaction motif		

**33**	in	36 AA		19	c.11388del (p.Asn3797fs)
34	in	28 AA	SET		
35	out		SET		
36	out		SET; post-SET		

ENST00000420124.4 (MANE select)

Exon #	Frame In/out	Ex size in amino acids	Domains	Reference #	NM_014727.3(*KMT2B*)
1	out		Menin binding domain; A+T hook DNA binding 1 and 2		
2	out				
3	out		A+T hook DNA binding		
**4**	in	38 AA			
5	out				
6	out		CXXC-type		
7	out		CXXC-type		
8	in	92 AA			
9	out				
**10**	in	33 AA			
11	in	38 AA	PHD-type 1		
12	in	49 AA	PHD-type 1; PHD-type 2		
13	in	32 AA	PHD-type 2		
14	out		PHD-type 2		
15	in	38 AA	PHD-type 3		
16	out		PHD-type 3, Bromodomain		
17	in	30 AA	Bromodomain		
18	in	35 AA	Bromodomain		
19	out		Bromodomain		
20	out				
21	out		C2HC pre-PHD type		
22	in	46 AA	C2HC pre-PHD type, PHD-type 4		
23	in	53 AA	PHD-type 4		
24	out		FYR N-terminal		
25	out		FYR N-terminal		
26	out		FYR N-terminal		
**27**	in	76 AA		1	c.5462dupC p.(Leu1822Thrfs*12)
				2	c.5636delG (p.Gly1879Valfs*16)
				3	c.5658delC (p.Ser1887Profs*8)
28	out				
**29**	in	30 AA			
30	out				
31	in	46 AA	FYR C-terminal		
32	in	25 AA	FYR C-terminal		
33	out		FYR C-terminal, WDR5 interaction motif		
**34**	in	36 AA		4	c.7614del (p.Thr2539fs)
35	in	28 AA	SET		
36	out		SET; S-adenosyl-L-methionine binding		
37	out		SET; S-adenosyl-L-methionine binding; post-SET		

ENST00000262189.11 (MANE select)

Exon #	Frame In/out	Ex size in amino acids	Domains	Reference #	NM_170606.3(*KMT2C*)
1	out		DNA binding, A.T hook		
2	out				
3	out				
**4**	in	67 AA		1	c.560C>G (p.Ser187Ter)
5	out		C2HC pre-PHD-type 1, degenerate		
6	out		C2HC pre-PHD-type 1, degenerate; PHD-type 1		
7	out		PHD-type 1		
8	out		PHD-type 2; PHD-type 3		
9	out		PHD-type 3		
10	out		PHD-type 3; DHHC; PHD-type 4		
11	out		PHD-type 4		
**12**	in	38 AA		2	c.1690A>T (p.Lys564Ter)
**13**	in	26 AA			
14	out				
**15**	in	40 AA			
**16**	in	39 AA		3	c.2693delC (p.Gly898Valfs*15)
17	in	34 AA	PHD-type 5		
18	in	35 AA	PHD-type 5		
19	out		PHD-type 5; PHD-type 6		
20	in	55 AA	PHD-type 6; PHD-type 7		
21	out		PHD-type 7		
**22**	in	22 AA		4	c.3462del (p.Leu1155_Val1156insTer)
**23**	in	71 AA		5	c.3599C>G (p.Ser1200Ter)
**24**	in	43 AA			
**25**	in	40 AA			
26	out				
27	out				
**28**	in	35 AA		6	c.4299_4307del (p.Asp1433_Ser1435del)
**29**	in	43 AA		7	c.4441C>T (p.Arg1481Ter)
**30**	in	11 AA			
**31**	in	40 AA		8	c.4629delinsCC (p.Thr1545fs)
32	out				
33	out		High mobility group box domain		
34	out		High mobility group box domain		
35	in	61 AA	High mobility group box domain		
36	in	628 AA			
37	out				
38	out				
39	out				
40	out				
41	out				
42	out				
43	in	570 AA			
**44**	in	70 AA		9	c.11548C>T (p.Arg3850Ter)
45	out				

46	out				
47	out				
48	out				
49	out				
50	out				
**51**	in	36 AA			
52	out		C2HC pre-PHD-type 2; PHD-type 8; FYR N-ter, FYR C-ter		
53	out		FYR C-ter		
54	out		FYR C-ter; WRD5 interaction motif		
55	out		SET		
56	in	39 AA	SET		
57	out		SET; S-adenosyl-L-methionine binding site		
58	out		SET		
59	out		SET, Post-SET		

ENST00000301067.12 (MANE select)

Exon #	Frame In/out	Ex size in amino acids	Domains	Reference #	NM_003482.4(*KMT2D*)
1	out				
2	out				
3	out				
4	out		C2HC pre PHD-type 1, degenerate		
5	out		C2HC pre PHD-type 1, degenerate; PHD-type 1		
6	out		PHD-type 1		
7	out		PHD-type 2; PHD-type 3		
8	out		PHD-type 3		
9	out		PHD-type 3		
10	out				
**11**	in	513 AA		1	c.1300del (p.Pro433_Leu434insTer)
				2	c.1301del (p.Leu434fs)
				3	c.1329_1332del (p.Pro444fs)
				4	c.1345_1346del (p.Leu449fs)
				5	c.1363G>T (p.Glu455Ter)
				6	c.1395dup (p.Arg466fs)
				7	c.1468_1471del (p.Glu490fs)
				8	c.1491_1492del (p.Pro497_Pro498insTer)
				9	c.1529C>G (p.Ser510Ter)
				10	c.1634del (p.Leu545fs)
				11	c.1748del (p.Pro583fs)
				12	c.1762_1763del (p.Ser588fs)
				13	c.1769dup (p.Met590fs)
				14	c.1813G>T (p.Glu605Ter)
				15	c.1825del (p.Ser609fs)
				16	c.1940del (p.Pro647fs)
				17	c.1966del (p.Leu656fs)
				18	c.1967del (p.Leu656fs)
				19	c.2091dup (p.Thr698fs)
				20	c.2110_2113delinsTC (p.Asp704fs)
				21	c.2164del (p.Glu722fs)
				22	c.2173_2174del (p.Leu725fs)
				23	c.2263dup (p.Arg755fs)
				24	c.2317dup (p.Gln773fs)
				25	c.2425dup (p.Gln809fs)
				26	c.2488G>T (p.Glu830Ter)
				27	c.2533del (p.Arg845fs)
				28	c.2546C>A (p.Ser849Ter)
				29	c.2578_2579del (p.Leu860fs)
				30	c.2579del (p.Leu860fs)
				31	c.2713del (p.Glu905fs)
				32	c.2760_2782del (p.Gly921fs)
				33	c.2782C>T (p.Gln928Ter)
12	out				
**13**	in	38 AA		34	c.3968dup (p.Gly1323_Arg1324insTer)
14	in	37 AA	PHD-type 4		
15	in	35 AA	PHD-type 4		
16	out		PHD-type 4; PHD-type 5		

17	out		PHD-type 5; PHD-type 6		
18	out		PHD-type 6		
**19**	in	16 AA		35	c.4710del (p.Glu1571fs)
				36	c.4739del (p.Pro1580fs)
**20**	in	74 AA		37	c.4762G>T (p.Glu1588Ter)
				38	c.4812dup (p.Met1605fs)
				39	c.4843C>T (p.Arg1615Ter)
				40	c.4941del (p.Asp1648fs)
**21**	in	40 AA		41	c.4981del (p.Glu1661fs)
				42	c.4981dup (p.Glu1661fs)
				43	c.4987G>T (p.Glu1663Ter)
				44	c.5058dup (p.Arg1687fs)
**22**	in	35 AA		45	c.5104C>T (p.Arg1702Ter)
				46	c.5124_5125del (p.Arg1709fs)
				47	c.5135_5136del (p.Lys1712fs)
				48	c.5149C>T (p.Gln1717Ter)
				49	c.5166del (p.Ser1722fs)
				50	c.5185G>T (p.Glu1729Ter)
23	out				
24	out				
**25**	in	22 AA		51	c.5526del (p.Asp1842fs)
**26**	in	37 AA			
**27**	in	46 AA			
28	out				
29	out		High mobility group box domain		
30	out		High mobility group box domain		
31	in	17 AA	High mobility group box domain		
32	in	604 AA	High mobility group box domain		
33	in	61 AA	LxxLL motif 1		
34	out				
35	out		LxxLL motif 2		
36	out				
37	out				
38	out				
39	out				
40	in	930 AA	LxxLL motif 3; 4; 5		
**41**	in	47 AA		52	c.13543dup (p.Arg4515fs)
				53	c.13579A>T (p.Lys4527Ter)
				54	c.13606C>T (p.Arg4536Ter)
				55	c.13650del (p.Leu4551fs)
				56	c.13652_13660del (p.Leu4551_Gln4554delinsTer)
**42**	in	56 AA		57	c.13780del (p.Ala4594fs)
				58	c.13818C>G (p.Tyr4606Ter)
43	out				
44	out				
45	out				
46	out				
47	out				
48	out				

49	out		LxxLL motif 6; C2HC pre-PHD-type 2; PHD-type 7; FYR N-terminal; FYR C-terminal		
50	out				
51	out		WRD5 interaction motif		
52	out		SET		
53	out		SET; S-adenosyl-L-methionine binding site		
54	out		SET		
55	out		SET; post-SET		

ENST00000311117.8 (MANE select)

Exon #	Frame In/out	Ex size in amino acids	Domains	Reference #	NM_182931.3(*KMT2E*)
1	out				
2	out				
3	out				
4	out				
5	out		HCF1-binding motif; PHD-type		
6	in	27 AA	PHD-type		
7	out				
8	out				
**9**	in	13 AA			
10	in	77 AA	SET		
11	out		SET		
12	out		SET		
13	out		SET		
14	out				
**15**	in	33 AA		1	c.1646_1650del (p.Ile549fs)
				2	c.1688C>G (p.Ser563Ter)
				3	c.1785_1786del (p.Arg597fs)
**16**	in	55 AA		4	c.1776_1780del (p.Lys593fs)
				5	c.1812delG (p.Ile605Serfs*41)
**17**	in	103 AA		6	c.2051_2052dup (p.Glu685Ter)
				7	c.2107G>T (p.Glu703Ter)
				8	c.2116dupA (p.Thr706Asnfs*9)
				9	c.2164_2167del (p.Lys722fs)
**18**	in	85 AA		10	c.2261del (p.Leu753_Ser754insTer)
				11	c.2337C>A (p.Tyr779Ter)
				12	c.2881_2882del (p.Lys961fs)
19	out				
20	out				
**21**	in	19 AA		13	c.2866dupA (p.Ser956Lysfs*11)
**22**	in	188 AA		14	c.2936del (p.Leu979fs)
				15	c.2941_2944delCCTT (p.Pro981Leufs*6)
				16	c.2960_2964del (p.Asn986_Leu987insTer)
				17	c.3034C>T (p.Gln1012Ter)
				18	c.3070C>T (p.Gln1024Ter)
				19	c.3198_3234del (p.Trp1067fs)
				20	c.3853dup (p.Ser1285fs)
23	out				
24	out				
**25**	in	42 AA		21	c.3908C>G (p.Ser1303Ter)

				22	c.3917dup (p.Pro1307fs)
26	out				
27	out				

ENST00000262519.14 (MANE select)

Exon #	Frame In/out	Ex size in amino acids	Domains	Reference #	NM_014712.3(*SETD1A*)
1	out				
2	out				
**3**	in	32 AA		1	c.214del (p.Arg72fs)
4	out		RNA recognition motif		
5	out				
6	out				
7	out				
8	in	262 AA		2	c.2121dupC (p.Gly708Argfs*117)
**9**	in	59 AA		3	c.2512C>T (p.Gln838Ter)
				4	c.2569G>T (p.Glu857Ter)
10	out				
11	out				
12	out				
**13**	in	114 AA			
14	in	350 AA	HCF1-binding motif; ASH2L, RBBP5, WRD5 interaction region		
15	out		ASH2L, RBBP5, WRD5 interaction region		
16	in	37 AA	ASH2L, RBBP5, WRD5 interaction region		
17	in	40 AA	SET		
18	in	46 AA	SET		
19	out		SET; post-SET		

ENST00000604567.6 (MANE select)

Exon #	Frame In/out	Ex size in amino acids	Domains	Reference #	NM_001353345.2(*SETD1B*)
1	out				
2	out				
**3**	in	33 AA			
4	out		RNA recognition motif		
5	out				
6	in	411 AA			
7	in	275 AA			
8	out				
9	out				
10	out				
**11**	in	164 AA		1	c.3901C>T (p.Arg1301Ter)
12	in	420 AA	N-SET		
13	out		N-SET, WRD5 interaction motif		
14	in	44 AA	N-SET		
15	in	40 AA	SET		
16	in	46 AA	SET		
17	in	58 AA	SET; post-SET		

ENST00000392403.8 (MANE select)

Exon #	Frame In/out	Ex size in amino acids	Functional domains	Reference #	NM_018489.3(*ASH1L*)
1	out				
2	out				
3	out		A+T hook 1, 2		
**4**	in	34 AA		1	c.5081dupC (p.Thr1695Asnfs*18)
5	out		A+T hook 3		
**6**	in	60 AA			
7	out				
8	out				
9	out				
10	out		AWS		
11	in	69 AA	AWS; SET		
12	in	49 AA	SET		
13	out		SET; post-SET		
14	in	55 AA	post-SET		
15	out				
16	out				
**17**	in	51 AA		2	c.7261C>T (p.Arg2421Ter)
18	out		Bromodomain		
19	out		Bromodomain		
20	in	83 AA	Bromodomain; PHD-type		
21	in	29 AA	PHD-type		
22	out		BAH		
23	out		BAH		
24	out		BAH		
25	out		BAH		
26	out				
27	out				
28	out				
