# Supplementary material for: Treatability of the KMT2-Associated Neurodevelopmental Disorders Using Antisense Oligonucleotide-Based Treatments
Source: Hum Mutat. 2024 May 29;2024:9933129. doi: 10.1155/2024/9933129 (PMC11925151; doi:10.1155/2024/9933129)
Supplement: Supplementary 2 — Supplementary methods: annotation of Supplementary Table S1 and details of the variant evaluation procedure. It includes Supplementary Table S2: summary of the analyzed published literature reporting phenotypic characteristics for the KMT2-associated disorders. [file 9933129.f2.docx]

**Supplementary methods**

**Supplementary Table S1:** List of (likely) pathogenic variants, obtained from databases (see Methods in main text) and literature search, amenable to an exon skipping approach. Supplementary Table S1 is supplied as a separate Excel file. The tabs were divided according to the gene name.

Table annotation

| Cell name | Description |
| --- | --- |
| NM_ | Identifier for the gene's MANE (Matched Annotation from the NCBI and EMBL-EBI ) Select transcript |
| Exon # | Exon number based on the MANE select transcript, the numeration includes non-coding exons. Exons suitable for exon skipping are bolded to match Figure 3 |
| Frame in/out | Exon frame, refers to whether or not the exon is divisible by 3 |
| Ex size in amino acids | Exon size is defined by the number of encoded amino acids |
| Domains | Functional domains encoded by the sequence contained in the exon |
| Reference # | The number assigned to the reported amenable variants. The enumeration is in ascending order from 5' to 3' |
| Variants | Variants were reported based on the GRCh38/hg38 reference genome and the MANE Select transcript for each gene using the HGVS nomenclature |

Variant evaluation procedure:

1. Exon number, frame, and size were determined using the Ensembl genome browser. Only the protein-coding transcript corresponding to the MANE Select transcript was considered.
2. Protein information regarding domains and features was obtained from UniProt.
3. Out-of-frame exons and in-frame exons encoding functional domains were excluded.
4. Variants were screened from the ClinVar, HGMD, and LOVD databases and considered for amenability to exon-skipping. Only (likely) pathogenic variants with a clearly reported phenotype were considered.
5. Additionally, if there is evidence that skipping an exon will cause the disease in question, they were excluded from the analysis. This is for example the case when pathogenic variants disrupt splicing, leading to exon-skipping themselves. Only in the presence of such variants, identified in individuals with a phenotype matching the *KMT2*-associated NDDs, and confirmed by functional testing, the exons would be excluded from consideration. In the absence of evidence regarding the pathogenic effect of skipping, larger (>10% of the encoded protein) in-frame exons encoding disordered regions, were also kept for consideration.

The detailed procedure of variant selection for exon skipping was described elsewhere (Zardetto *et al.*, accepted).

**Supplementary Table S2**: Summary of the published literature reporting patient phenotypes for the *KMT2*-associated disorders which was used to perform our analysis (Figure 1). This list does not include all cases reported for these genes, it is only a reference for the studies included here.

| Gene | Cases | First author, year published | DOI |
| --- | --- | --- | --- |
| *KMT2A* | 52 | Lin *et al.*, 2023 | 10.3389/fgene.2023.1085210 |
|  | 104 | Sheppard *et al.*, 2021 | 10.1002/AJMG.A.62124 |
|  | 16 | Lin *et al.*, 2018 | 10.1186/s13023-018-0909-0 |
|  | 33 | Baer *et al.*, 2018 | 10.1111/cge.13254 |
| *KMT2B* | 53 | Cif *et al.*, 2020 | 10.1093/brain/awaa304 |
|  | 28 | Meyer *et al.*, 2017 | 10.1038/ng.3740 |
|  | 13 | Carecchio *et al.*, 2019 | 10.1002/mds.27771 |
|  | 10 | Zech *et al.*, 2016 | 10.1016/J.AJHG.2016.10.010 |
|  | 5 | Dai *et al.*, 2019 | 10.1016/j.parkreldis.2018.08.021  10.3760/cma.j.issn.0578-1310.2019.07.015 |
|  | 4 | Kawarai *et al.*, 2018 | 10.1016/j.parkreldis.2018.03.022 |
|  | 4 | Dafsari *et al.*, 2019 | 10.1038/s10038-019-0625-1 |
|  | 2 | Ma *et al.*, 2019 | 10.3389/fneur.2019.00729 |
|  | 3 | Zech *et al.*, 2017 | 10.1002/mds.27026 |
|  | 1 | Zech *et al.*, 2017 | 10.1007/s10048-017-0521-9 |
|  | 1 | Baizabal-Carvallo and Alonso-Juarez, 2018 | 10.1016/j.parkreldis.2018.01.016 |
|  | 1 | Faundes *et al.*, 2018 | 10.1016/j.ajhg.2017.11.013 |
|  | 1 | Hackenberg *et al.*, 2018 | 10.1055/s-0038-1661343 |
|  | 1 | Zhao *et al.*, 2018 | 10.1016/j.bbadis.2018.03.020 |
|  | 1 | Brás *et al.*, 2019 | 10.1212/WNL.0000000000007469 |
|  | 1 | Klein *et al.*, 2019 | 10.1016/j.parkreldis.2019.03.018 |
|  | 1 | Miyata *et al.*, 2019 | 10.1111/ncn3.12334 |
|  | 1 | Zhou *et al.*, 2019 | 10.1016/j.parkreldis.2018.09.020 |
|  | 1 | Cao *et al.*, 2020 | 10.1002/mdc3.12862 |
|  | 1 | Mun *et al.*, 2020 | 10.14802/jmd.19087 |
| *KMT2C* | 1 | Siano *et al.*, 2022 | 10.3390/pediatric14010019 |
|  | 1 | Schoch *et al.*, 2020 | 10.1038/s41436-020-0781-x |
|  | 3 | Faundes *et al.*, 2018 | 10.1016/j.ajhg.2017.11.013 |
|  | 5 | Koemans *et al.*, 2017 | 10.1371/journal.pgen.1006864 |
|  | 1 | Kleefstra *et al.*, 2012 | 10.1016/j.ajhg.2012.05.003 |
| *KMT2D* | 15 | Di Candia *et al.*, 2022 | 10.1007/s00431-021-04108-w |
|  | 18 | Lindsley *et al.*, 2016 | 10.1016/j.jaci.2015.06.002 |
|  | 12 | Cheon *et al.*, 2015 | 10.3345/kjp.2015.58.9.317 |
|  | 20 | Banka *et al.*, 2012 | 10.1038/ejhg.2011.220 |
|  | 20 | Schrander-Stumpel *et al.*, 2005 | 10.1002/ajmg.a.30331 |
|  | 27 | White *et al.*, 2004 | 10.1002/ajmg.a.20674 |
|  | 247 | Matsumoto *et al.*, 2003 | 10.1002/ajmg.c.10020 |
| *KMT2E* | 18 | Velmans *et al.*, 2022 | 10.1136/jmedgenet-2020-107470 |
|  | 34 | O’Donnell-Luria *et al.*, 2018 | 10.1016/J.AJHG.2019.03.021 |
| *SETD1A* | 15 | Kummeling *et al.*, 2021 | 10.1038/s41380-020-0725-5 |
| *SETD1B* | 36 | Weerts *et al.*, 2021 | 10.1038/s41436-021-01246-2 |
|  | 4 | Roston *et al.*, 2021 | 10.1136/jmedgenet-2019-106756 |
|  | 3 | Krzyzewska *et al.*, 2019 | 10.1186/s13148-019-0749-3 |
|  | 1 | Den *et al.*, 2019 | 10.1038/s10038-019-0617-1 |
|  | 1 | Hiraide *et al.*, 2019 | 10.1002/epi4.12339 |
|  | 1 | Hiraide *et al.*, 2018 | 10.1007/s00439-017-1863-y |
| *ASH1L* | 1 | Okamoto et al., 2017 | 10.1002/ajmg.a.38193 |
|  | 5 | Stessman et al., 2017 | 10.1038/ng.3792 |
|  | 1 | Wang et al., 2016 | 10.1038/ncomms13316 |
|  | 1 | de Ligt et al., 2012 | 10.1056/NEJMoa1206524 |
